# Giant cell tumor of the cervical spine treated by carbon ion radiotherapy: A case report

**DOI:** 10.1097/MD.0000000000027393

**Published:** 2021-10-15

**Authors:** Tomohiko Sakuda, Taisuke Furuta, Tomoaki Okimoto, Nobuo Adachi

**Affiliations:** a Department of Orthopaedic Surgery, Graduate School of Biomedical and Health Sciences, Hiroshima University, 1-2-3 Kasumi, Minami-ku, Hiroshima, Japan; b Hyogo Ion Beam Medical Center, 1-2-1 Kouto, Shingu-cho, Tatsuno, Hyogo, Japan.

**Keywords:** carbon ion beam, giant cell tumor, recurrence

## Abstract

**Introduction:**

Giant cell tumor (GCT) of the bone is a benign–malignant intermediate tumor with locally destructive growth and a relatively high local recurrence rate. Neurological symptoms may develop in patients with GCT of the spine, and surgical treatment is prioritized in cases where resection is possible. However, the local recurrence rate of GCT of the bone is higher than that of GCT at other sites owing to the associated surgical challenges, and treatment is often difficult. No study to date has reported long-term remission of recurrent tumors for more than 5 years by treatment with carbon ion beam radiotherapy after resection of GCT of the cervical spine.

**Patient concerns:**

A 14-year-old boy who experienced recurrence after surgery for GCT of the cervical spine.

**Diagnosis:**

The patient presented with cervical pain, and computed tomography revealed a mass of the C2 vertebral body. He underwent surgery for tumor resection and autologous bone grafting, and the final pathological diagnosis was GCT. The transplanted bone exhibited gradual progression of resorption, and recurrent tumors were observed on computed tomography and magnetic resonance imaging 1 year and 4 months after surgery.

**Interventions:**

The patient was started on denosumab at 15 years of age and received carbon ion beam therapy with 70.4 Gy administered in 32 sessions over 7 weeks.

**Outcomes:**

No progressive tumor growth was observed, there were no neurological symptoms such as paralysis or pain were noted, and the patient was in remission for 5 years after irradiation.

**Conclusion:**

These findings suggest that carbon ion radiotherapy is a safe and effective therapeutic option for patients with recurrent GCT of the cervical spine.

## 1 Introduction

Giant cell tumor (GCT) of the bone is a benign–malignant intermediate tumor that exhibits locally destructive growth and a relatively high local recurrence rate.^[1]^ GCT usually affects young adults aged 20 to 30 years after epiphyseal closure^[1]^ but is occasionally detected in children before epiphyseal closure. Furthermore, patients with spinal involvement may present with neurological symptoms.^[2]^ Surgical treatment is prioritized if resection of GCT is possible.^[1]^ However, the local recurrence rate of spinal GCT is higher than that of GCT at other sites owing to challenges associated with the surgical procedure,^[3]^ and treatment is often difficult.^[2–6]^ Herein, we report the case of a patient who experienced recurrence 1 year and 4 months after surgery for GCT of the cervical spine and was successfully treated by carbon ion radiotherapy, illustrating the potential utility of this approach for the treatment of similar cases.

## 2 Case report

**Figure 1:**
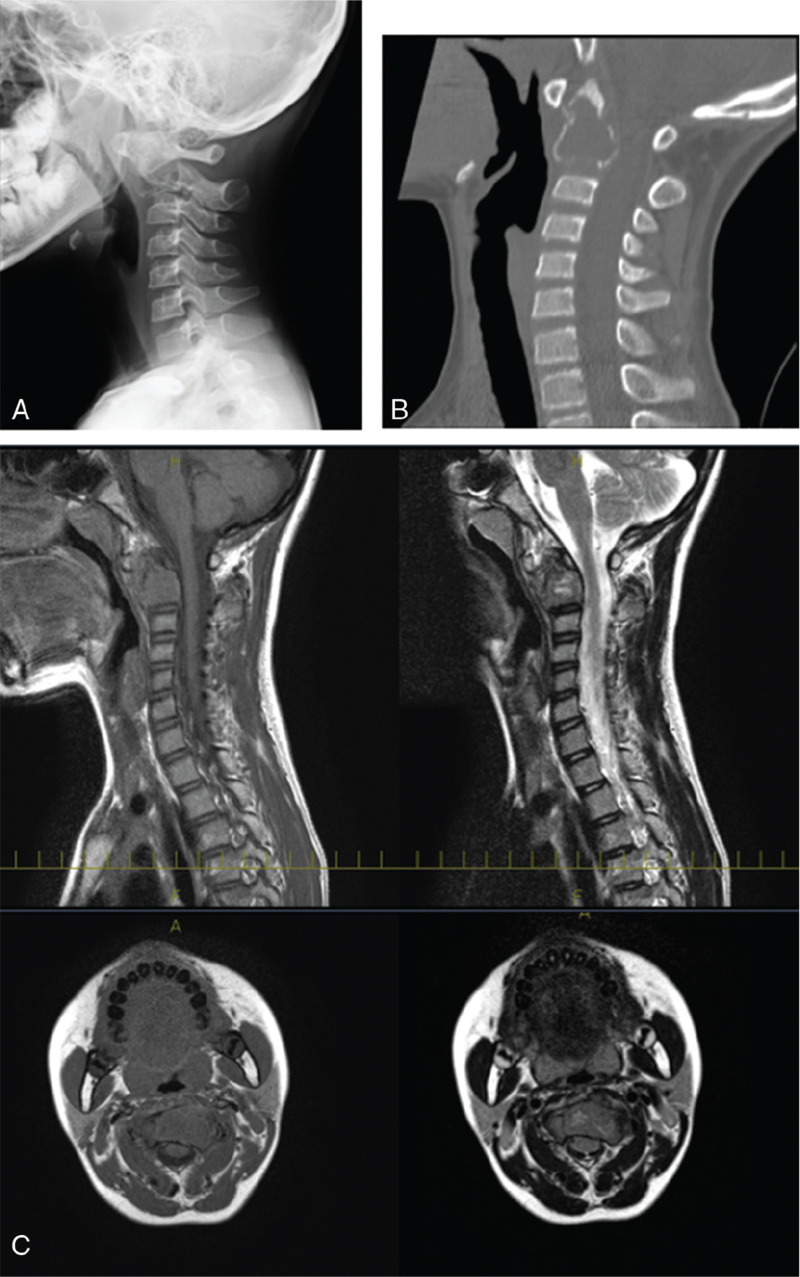
Radiological (A), computed tomography (B), and magnetic resonance (C) images during the first visit of the patient to our hospital (A). (A, B) Plain radiography and computed tomography show a radiolucent shadow and a mild osteolytic lesion of the C2 vertebral body. (C) Magnetic resonance imaging shows a mass of the C2 vertebral body, with low signal intensity on T1-weighted images and heterogeneous high signal intensity on T2-weighted images.

A 14-year-old boy developed cervical pain at 12 years of age after being pushed on his back by a friend and consulted a doctor. Plain radiography revealed a radiolucent shadow and a mildly osteolytic lesion of the C2 vertebral body (Fig. 1A). A bone tumor at the C2 vertebral body was suspected, and a pharyngeal biopsy was performed for confirmation. He was diagnosed with juvenile xanthogranuloma and underwent posterior spinal fusion at the C1 to C3 level, followed by chemotherapy. However, the tumor continued to grow and the patient was referred to our hospital at 14 years of age.

On examination, mild swelling was noted at the base of the neck; however, he did not exhibit erythema, local heat, or constitutional symptoms such as fever, weight loss, and malaise. Computed tomography confirmed the previous radiographic findings (Fig. 1B), and magnetic resonance imaging revealed a mass at the C2 vertebral body, with low signal intensity on T1-weighted imaging and heterogeneously high signal intensity on T2-weighted imaging (Fig. 1C).

**Figure 2:**
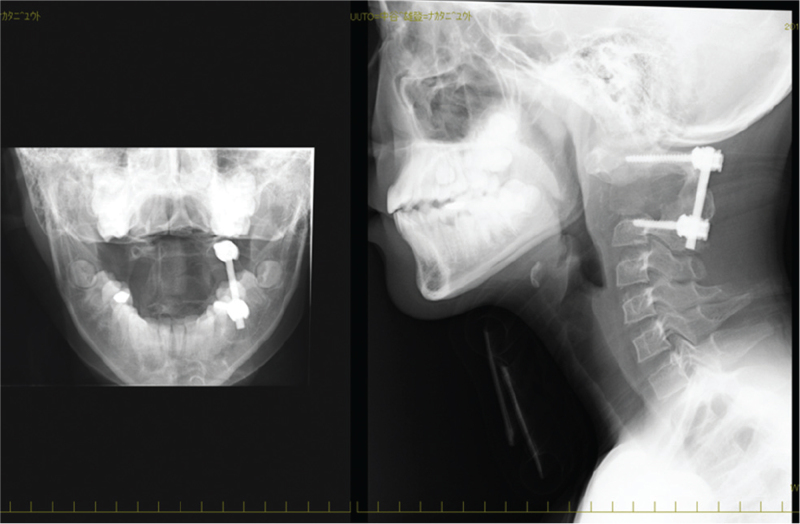
Radiological image after surgical treatment shows fixed from C1 to C3 on the left side.

After being informed of the potential risks associated with the intervention, the patient underwent surgery for tumor resection and autologous bone grafting. Exploration of the vertebral artery and the right C2 and C3 nerve roots revealed an extraosseous brownish tumor piercing the right lateral cortex of the C2 vertebral body. The tumor was excised piecemeal. The tumor at the C2 vertebral body was also excised to the greatest extent possible. The C2/C3 disc was removed, and the anterior tumor was excised to expose the dura. The tumor in the dental process and the tumor on the left side were also excised. A 3-cm wide full-thickness bone harvested from the ilium was formed and transplanted between the C1 and C3 vertebral bodies (Fig. 2). The histological assessment revealed round mononuclear stromal cells and osteoclast-like polynuclear giant cells in the excised tumor and the final pathological diagnosis was GCT.

**Figure 3:**
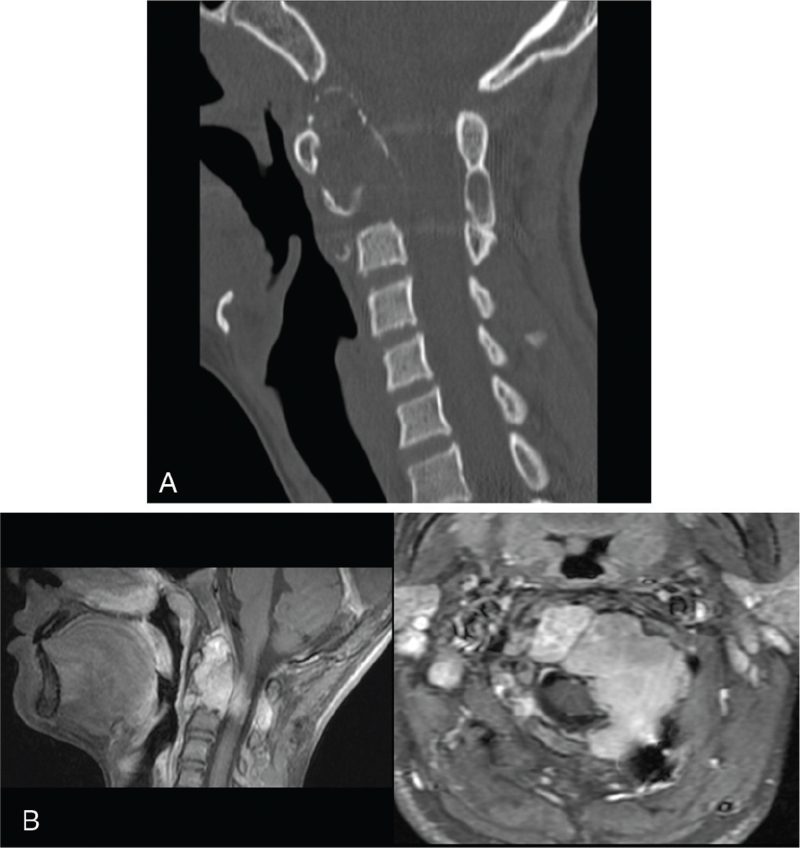
Computed tomography (A) and magnetic resonance (B) images of the cervical spine 1 yr after the surgery. Magnetic resonance images revealed gradual resorption of the transplanted bone and recurrent tumors.

**Figure 4:**
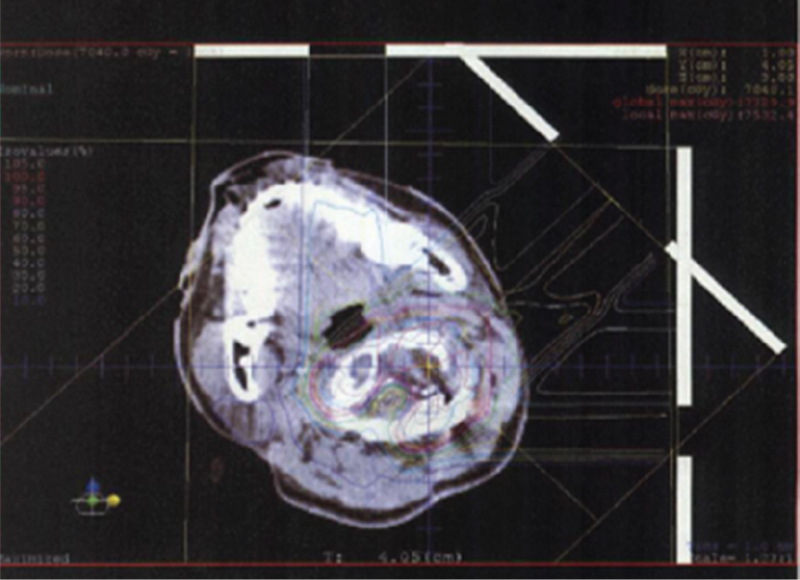
Representative image of dose distribution of carbon ion radiotherapy administered to the patient.

**Figure 5:**
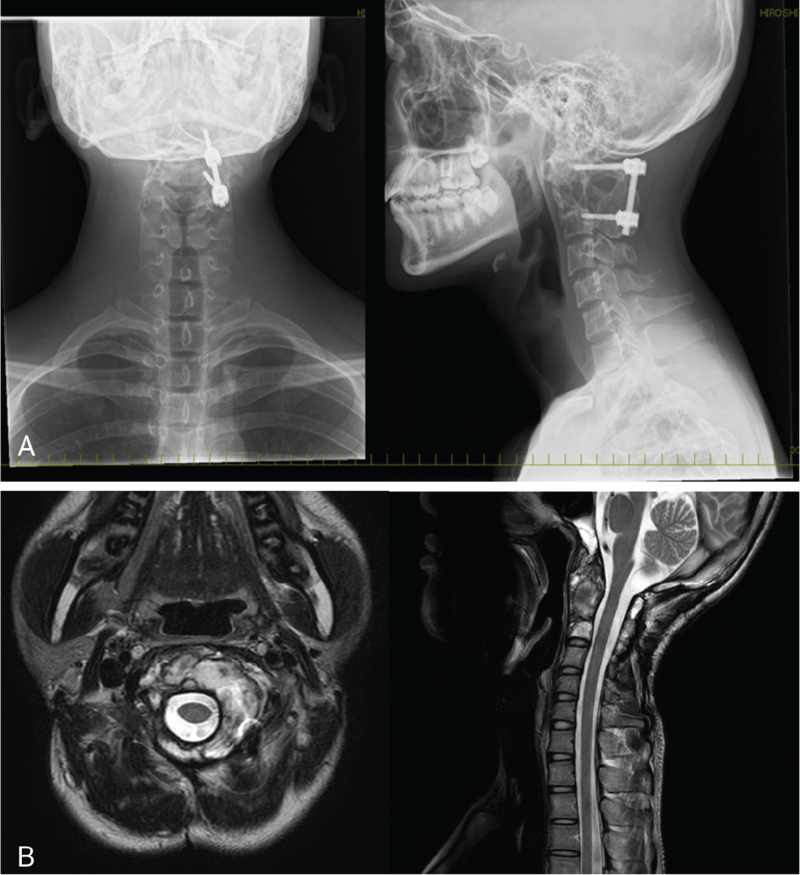
Radiological (A) and magnetic resonance (B) images of the cervical spine show the absence of tumor growth 5 yrs after carbon ion radiotherapy.

Postoperative follow-up was performed at regular intervals without additional adjuvant therapy. The transplanted bone exhibited gradual resorption, and the number of recurrent tumors detected on computed tomography and magnetic resonance imaging 1 year and 4 months after surgery increased (Fig. 3A, B). Therefore, carbon ion radiotherapy was planned (Fig. 4) at the Hyogo Ion Beam Medical Center in Japan, leading to the global initiation of carbon ion radiotherapy. Due to an interval of 5 months between the planning and initiation of carbon ion radiotherapy, the patient was started on denosumab as a palliative treatment during that period at 15 years of age. Denosumab (RANMARK 120 mg; Daiichi-Sankyo, Tokyo, Japan) was administered subcutaneously on days 1, 8, 15, and 29, and every 4 weeks after the first month. Following 5 months of denosumab treatment (with denosumab being administered a total of 9 times), the patient received carbon ion beam therapy with 70.4 Gy administered in 32 sessions over 7 weeks. Mild dermatitis, which occurred in the irradiated area of the neck, was successfully treated with an ointment. Follow-up computed tomography or magnetic resonance imaging was performed every 3 months after carbon ion therapy; however the imaging studies revealed no evidence of further tumor growth and showed that the tumor size had remained unchanged (Fig. 5A, B). The patient did not exhibit any tumor-related symptoms such as paralysis or pain and was in remission for 5 years after radiotherapy.

## 3 Discussion

In the present case, the postoperative histopathological findings of a mixture of round or oval mononuclear stromal cells and osteoclast-like polynuclear giant cells in the resected specimen confirmed the diagnosis of GCT.^[1,2]^

Surgery is the first choice of treatment for GCT of the bone. However, some studies have reported local recurrence rates ranging approximately 10% to 30% even after complete curettage.^[1,2]^ Furthermore, compared with GCT diagnosed at other sites, the local recurrence of GCT of the spine diagnosed during spinal development is suggested to be even higher, which is related to surgical challenges, and treatment is often difficult. Another issue associated with the GCT of the spine is that the spinal development might be accompanied by neurological symptoms.

Treatment with denosumab, a receptor activator for nuclear factor κB ligand inhibitor, is considered for patients with difficult-to-resect GCT of the bone and in cases where nerve paralysis after resection is a concern.^[7–9]^ Denosumab is administered to patients with unresectable tumors and to those where major dysfunction due to resection is expected.^[7–9]^ Although a study reported that the recurrence of GCT of the cervical spine in a patient could be locally controlled in the short term using denosumab,^[10]^ the long-term prognosis with this approach remains unknown. The patient was a teenager undergoing bone growth, and the recommended administration period for denosumab has not been established. Furthermore, radical surgery for recurrent GCT of the spine is considered even more difficult; therefore, heavy ion radiotherapy was considered in the present case.

The general indications for heavy ion radiotherapy include localized unresectable malignant bone and soft tissue tumors. Tumors with an intermediate malignant presentation that are difficult to cure by surgery alone and cases in which patients are medically unfit for surgery are also target treatment groups for heavy ion therapy. Compared with X-ray/photon radiotherapy, the physical characteristics of heavy ion radiotherapy produce a conformal radiation dose distribution in the target. The defined beam range allows delivery of higher radiation doses than X-ray radiotherapy to cancerous lesions while minimizing the range of irradiation of surrounding normal tissues; thus, the risk of toxicity can be reduced.^[11,12]^ The evaluation of equivalent physical doses delivered by X-ray and particle radiotherapy in biological experiments revealed that the therapeutic effect that is, relative biological effectiveness of proton beams is slightly stronger (approximately 1.1 times) than that of X-rays.^[12,13]^ Furthermore, the relative biological effectiveness of carbon ion beams, a type of heavy ion radiotherapy, is approximately 3 times stronger than that of X-rays.^[12,13]^ Therefore, the radiation dose to the organ at risk in carbon ion radiotherapy is lower than that in X-ray and proton ion radiotherapy, which facilitates reduction of side effects. Carbon ion radiotherapy is particularly effective in the treatment of cancers that are less responsive to X-ray radiotherapy.^[11,12]^

In a study on radiotherapy for tumors of the skeletal system, among 8 patients with GCT, 1 patient experienced local failure, 1 patient experienced distant failure, and all patients were alive.^[14]^ The 5-year rate of local control was 83% in patients with GCT treated by proton beam radiotherapy.^[14]^ The present patient was treated with carbon ion radiotherapy. Despite the lack of large-scale studies, in patients with GCT, a higher disease control rate for patients with GCT can be expected with heavy ion (carbon ion) radiotherapy than that with proton beam radiotherapy. To the best of our knowledge, the long-term remission of recurrent tumors for more than 5 years by treatment with heavy ion (carbon ion beam) radiotherapy for recurrent tumor after resection of GCT of the cervical spine has not been reported yet. Given his young age, the present patient should be evaluated with regular follow-up.

In conclusion, the present case illustrates that heavy ion radiotherapy may be considered as an option for patients with difficult-to-treat or recurrent spinal GCT.

## Author contributions

All authors were responsible for the study concepts. Data acquisition, analysis, and interpretation were undertaken by TS, TF, TO, and NA. Drafting of the manuscript was the responsibility of TS, TF, TO, and NA. The final version of the manuscript to be published was approved by TS, TF, TO, and NA, and agreement on being accountable for all aspects of the work was reached upon by TS, TF, TO, and NA.

**Conceptualization:** Tomohiko Sakuda, Taisuke Furuta, Tomoaki Okimoto.

**Data curation:** Tomohiko Sakuda, Taisuke Furuta, Tomoaki Okimoto.

**Formal analysis:** Tomohiko Sakuda, Taisuke Furuta.

**Investigation:** Tomohiko Sakuda, Taisuke Furuta.

**Methodology:** Tomohiko Sakuda, Taisuke Furuta.

**Project administration:** Tomohiko Sakuda, Taisuke Furuta, Tomoaki Okimoto.

**Resources:** Tomohiko Sakuda, Taisuke Furuta.

**Software:** Tomohiko Sakuda.

**Supervision:** Tomohiko Sakuda, Taisuke Furuta, Tomoaki Okimoto, Nobuo Adachi.

**Validation:** Tomohiko Sakuda, Taisuke Furuta, Tomoaki Okimoto, Nobuo Adachi.

**Visualization:** Tomohiko Sakuda, Taisuke Furuta, Tomoaki Okimoto, Nobuo Adachi.

**Writing – original draft:** Tomohiko Sakuda.

**Writing – review & editing:** Tomohiko Sakuda, Taisuke Furuta, Tomoaki Okimoto, Nobuo Adachi.
